# Incidence of appendiceal neoplasms in appendectomy patients

**DOI:** 10.1186/s12893-023-02183-4

**Published:** 2023-09-21

**Authors:** Ricardo E. Núñez-Rocha, Felipe Girón, Lina Rodríguez, Daniela Camargo-Gómez, Carolina Restrepo-Bonilla, Rocío Del Pilar López Panqueva, Manuel Cadena, Ricardo Nassar, Gabriel E. Herrera-Almario, Juan David Hernández-Restrepo

**Affiliations:** 1 Hospital Universitario Fundación Santa Fe de Bogotá, Calle 119 No. 7-14, Bogotá, DC Colombia; 2 School of Medicine, Universidad de los Andes, Cundinamarca, Colombia; 3 Hospital Universitario San Ignacio, Bogotá, Colombia

**Keywords:** Appendiceal neoplasms, Appendectomy, Appendicitis, Neuroendocrine tumor, Appendiceal mucinous neoplasms, Sessile serrated adenomas

## Abstract

**Background:**

Non-operative management has been suggested as a therapy for uncomplicated appendicitis. Notwithstanding, the risk of missing an appendiceal tumor must be considered, being the surgical piece crucial to rule out neoplasms. Therefore, we aim to determine the incidence of appendiceal neoplasms in patients with acute appendicitis, tumor types and the importance of the anatomopathological study of the surgical piece.

**Study design:**

Retrospective study in which we described patients who underwent emergent appendectomy with histopathological findings of appendiceal neoplasms from January 2012 to September 2018. Descriptive analysis included demographic variables, diagnostic methods, and surgical techniques.

**Results:**

2993 patients diagnosed with acute appendicitis who underwent an emergency appendectomy. 64 neoplasms of the appendix were found with an incidence of 2,14%. 67.2% were women, the mean age was 46,4 years (± 19.5). The most frequent appendiceal neoplasms were neuroendocrine tumors (42,2%), followed by appendiceal mucinous neoplasms (35,9%), sessile serrated adenomas (18,8%), and adenocarcinomas (3,1%). In 89,1% of the cases, acute appendicitis was determined by imaging, and 14% of cases were suspected intraoperatively. Appendectomy was performed in 78,1% without additional procedures.

**Conclusions:**

Appendiceal tumors are rare and must be ruled out in patients with suspected acute appendicitis. The incidence of incidental neoplasms is higher in this study than in the previously reported series. This information must be included in decision-making when considering treatment options for acute appendicitis.

**Supplementary Information:**

The online version contains supplementary material available at 10.1186/s12893-023-02183-4.

## Introduction

Appendectomy has been widely accepted as first-line management for acute appendicitis in the absence of abscess formation or peritonitis [1–4]. However, recent studies randomized clinical trials have shown that in the short term, medical management with antibiotics is a safe alternative treatment option for cases of uncomplicated acute appendicitis [1, 5–7]. Nevertheless, the possibility of appendiceal neoplasms incidental diagnosis must be taken into account [4, 5, 8–10].

Tumors of the appendix are unusual entities, mainly diagnosed incidentally in the anatomopathological study of appendectomy pieces, and rarely suspected before or during a surgical procedure [5, 6, 8, 11–15]. Therefore, the study of the appendiceal sample is crucial both to diagnose the presence of neoplasms, as well as to determine their histological subtype [4, 10, 12, 15]. This information is useful for both establishing prognosis and outcomes, as well as for determining requirements for additional treatment [4, 10, 12, 15], accounting for tumor size, location, level of infiltration, and the resection margin status [12, 15]. The histological characteristics of appendiceal neoplasms are strong predictors of patients’ survival and must be included in the staging [5, 10, 16, 17]. Failure to detect appendiceal cancer delays diagnosis [6] which could lead to either an increase in the incidence of colon cancer [3, 18, 19] (mainly in patients over 40 years [5, 19, 20]) or to neoplastic disease due to perforation or rupture of a mucinous tumor (*Pseudomyxoma peritoneum*) [4, 21]. Despite tumors of the appendix being an infrequent diagnosis, the finding of these cases indicates the importance and need for the anatomopathological study of all the segments extracted during surgery [22].

These neoplasms correspond to less than 0,5% of all tumors of the gastrointestinal tract [23, 24], and are found in approximately 1% of the specimens from appendectomies [23–25]. Its incidence is low but has increased as reported by different international series [9–11]. Worldwide, it varies between 0,2% and 2,5% [5, 11, 25] (between 0,07% and 0,8% in Chile [11], 0,78% in Turkey [15], 0,9% in Spain [18], between 0,9% and 1,7% in the United States [6], 1,24% in Finland [5], 2,3% in Japan [9], and < 3% in Tunisia [16]). In Colombia, the incidence of tumors of the appendix is unknown. Therefore, we aimed to determine the incidence of appendiceal neoplasms in a cohort of patients with acute appendicitis, the histopathological types, the demographic factors of the patients, and to evaluate the importance of the routine anatomopathological study of the extracted surgical specimen.

## Methodology

After Institutional Review Board’s approval, a retrospective study composed of patients with diagnosed acute appendicitis who underwent emergency appendectomies at a tertiary referral hospital was conducted. Over 7,5 years between January 2012 and September 2018, 2993 clinical records were reviewed. Patients over 18 years old, and those who underwent appendectomy for acute appendicitis, were included. Patients with previously known diagnoses of an appendiceal tumor, those undergoing right hemicolectomy (RHC), and patients who underwent appendectomy for a different indication than acute appendicitis, were excluded. To clarify, individuals with an Alvarado rating ranging from 1 to 4 were discharged. Alvarado scoring ranging from 5 to 6, were sent to ultrasound examination and abdominal CT scans if needed. Individuals with an Alvarado rating of 7 to 10 were directly diagnosed with acute appendicitis and underwent appendectomy. Demographic variables (such as the age of presentation and gender), as well as diagnostic methods, surgical techniques (including the extension of the resection), and the histological type described in the pathology report, were analyzed using descriptive statistics. The follow-up on these patients was continued until the first postoperative control.

## Results

Of the 2993 samples of appendectomies analyzed, 64 neoplasms of the appendix were found (as shown in Table 1), representing an incidence of 2,14%. The demographic characteristics of the subjects are summarized in Table 2. 67,2% were women, and the mean age for men was 46,4 years (± 19.5). In 89,1% of the cases, the diagnosis of acute appendicitis was determined through imaging, with a suspicion of an appendiceal tumor in 12,5% of them. In 78,1% of the cases, appendectomy was performed without requiring additional interventions. 98,4% of the procedures performed were through a laparoscopic approach and there was no need for conversion. The reintervention and complications rates were 0%. Of the cases that were diagnosed with appendiceal neoplasms, 14% were suspected intraoperatively.

**Table 1:** Distribution of appendiceal neoplasms and degrees of differentiation (n = 64)

Variable	n(%)
**Benign**	**23(35.9)**
Mucinous neoplasm of appendix	23 (35.9)
Low grade of differentiation	20 (86.9)
High grade of differentiation	1 (4.3)
No degree of differentiation	2 (8.7)
Premalignant lesions	**12 (18.8)**
Sessile serrated adenoma	
High-grade dysplasia	1 (8.3)
No high-grade dysplasia	11 (91.6)
**Malignant**	**29 (45.3)**
Neuroendocrine tumor	27 (42.2)
Well-differentiated (G1)	25 (92.6)
Moderately differentiated (G2)	2 (7.4)
Adenocarcinoma	2 (3.1)
Well-differentiated	1 (0.5)
Poorly differentiated	1 (0.5)

**Table 2:** Demographic and clinical characteristics of the patients (n = 64)

Variable	n(%)
Female	43 (67.2)
Male	21 (32.8)
Age (average, SD)	46.4 (19.4)
**Diagnosis of acute appendicitis**	
Clinical (not imaging)	7 (10.9)
Imaging	57 (89.1)
Abdomen Ultrasound	9 (15.78)
Computed tomography of the abdomen	45 (78.9)
Magnetic resonance imaging of the abdomen	1(1.7)
Computed Tomography Urography	1 (1.7)
Positron emission tomography	1 (1.7)
**Suspicion of appendiceal neoplasm**	
Clinical	0 (0)
Imaging	8 (12.5)
Intraoperative	9 (14.1)
No suspicion of neoplasia	47 (73.4)
**Type of surgical procedure**	
Open appendectomy	1 (1.6)
Laparoscopic appendectomy	63(98.4)
**Extension of the resection**	
Appendectomy without other procedure	50 (78.1)
Appendectomy associated with another procedure	14 (21.9)

**Fig. 1:**
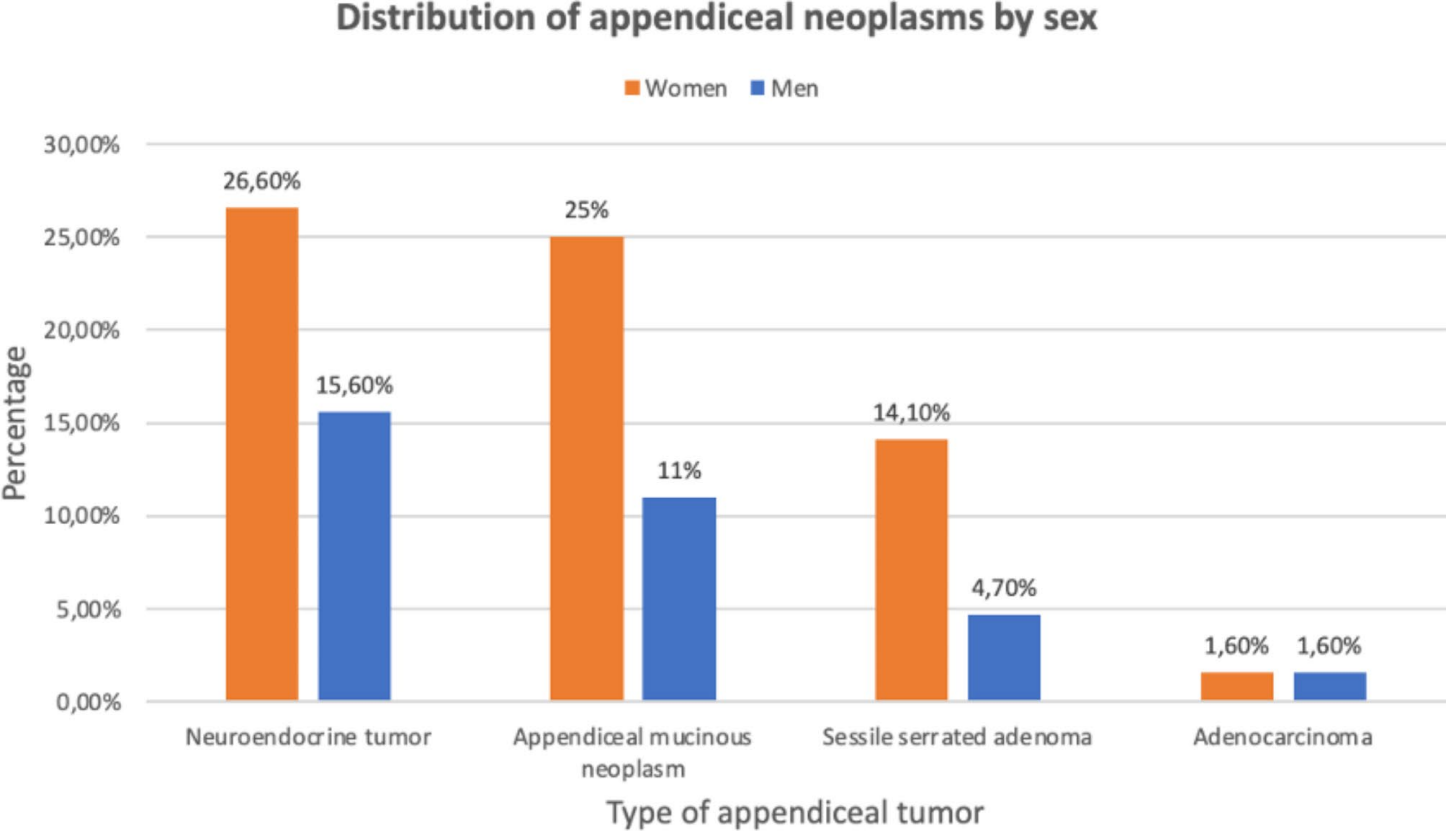
Distribution of appendiceal neoplasms by sex

The most frequent appendiceal neoplasm found was the neuroendocrine tumor (NET) reported in 27 patients (42,2%), of which 62,9% of the cases were women (Fig. 1), with an average age of presentation of 32.7 (± 12.3) years. In none of these patients, a tumor was suspected before the surgical procedure, and only in 2 cases (7,4%), there was an intraoperative suspicion of a tumor. According to the classification of the American Joint Committee of Cancer (AJCC) [27], well-differentiated tumors (G1) were found in 92,6% of the cases, while the remaining 7,4% were moderately differentiated (G2). 58.6% were tumors smaller than 1 cm, and only 2 specimens (6.9%) exceeded 2 cm in size (as shown in Table 3). In 89,7% of the cases, tumor-free resection margins were reported regarding malignant tumors.

**Table 3:** Histopathological characteristics of the malignant tumors of the appendix (n = 29)

Variable	Characteristic	n(%)
Size (centimeters)		
	< 1	17 (58.6)
	1–2	8 (27.6)
	> 2	2 (6.9)
	No data	2 (6.9)
**Parietal commitment**		
	Mucous membrane	0 (0)
	Submucosa	3 (10.3)
	Muscular	6 (20.7)
	Subserosa	9 (31.0)
	Serosa	3 (10.3)
	NA	8 (27.6)
**Commitment of the mesoappendix**		
	Yes	7 (24.1)
	No	2 (6.9)
	NA	20 (69)
**Lymphovascular invasion**		
	Yes	1 (3.4)
	No	23 (79.3)
	NA	5 (17.2)
**Resection margins**		
	Committed	1 (3.4)
	Free	26 (89.7)
	NA	2 (6.9)

The second most frequent tumor was mucinous neoplasm of the appendix (AMN) reported in 23 samples (35,9%), of which 69,6% were women (Fig. 1). The average overall age of presentation was 62.1 (± 17) years. Suspicion of neoplasm in the computed abdominal tomography was made in 7 cases (30,4%), mucocele of the appendix was suspected in 6 patients, and the presence of a mass in the cecum was suspected in 1 case. An appendiceal tumor was suspected during the surgical procedure in 7 patients (30,4%). 86,9% were AMNs with a low degree of differentiation. In 7 of the samples, mucin content was found, and also 7 presented perforations (as shown in Table 4). In 82,6% of the cases, tumor-free resection margins were reported.

**Table 4:** Histopathological characteristics of mucinous neoplasms of the appendix (AMN) (n = 23)

Variable	Characteristic	n(%)
**Mucin content**		
	Yes	7 (30.4)
	No	4 (17.4)
	No data	12 (52.2)
**Perforated appendicitis**		
	Yes	7 (30.4)
	No	4 (17.4)
	No data	12 (52.2)
**Section edges**		
	Committed	2 (8.7)
	Free	19 (82.6)
	No data	2 (8.7)

The sessile serrated adenoma (SSA), considered a pre-malignant lesion, was the third most frequent appendiceal tumor in this series, reported in 12 cases (18,8%). The mean age was 46 (± 15.6) years. Of the 12 SSAs, 9 cases (75%) were women (Fig. 1), and the average age of presentation was 44.8 years (± 15,8). In none of the patients, a tumor was suspected before or during the surgical procedure. 91,6% were SSAs without high-grade dysplasia. In 58,3% of the cases, tumor-free resection margins were reported.

The lowest frequency neoplasm found in the series was adenocarcinoma present in only 2 specimens (3,1%). The ratio of women to men was 1:1, with an average age of presentation of 54 years. One of the cases was reported as a well-differentiated neoplasm, and the other was poorly differentiated. Neither of the tumors was suspected before the surgical procedure. In one case, non-tumor-free resection margins were reported. Table 5 summarizes the surgical approach, according to the histopathological report of the appendectomy specimen.

**Table 5:** Conduct suggested by the surgery group after the histopathological report (n = 64)

Variable	n(%)
**Discharge after surgery**	**11 (17.2)**
SSA without high grade dysplasia	6 (54.5)
Well-differentiated NET	4 (36.4)
AMN low grade	1 (9.1)
**Follow-up by surgery**	**16 (25)**
AMN low grade	7 (43.7)
NET well-differentiated	6 (37.5)
SSA without high grade dysplasia	1 (6.3)
SSA dysplasia with high grade	1 (6.3)
AMN high grade	1 (6.3)
**HCD indication**	**10 (15.6)**
NET well-differentiated	5 (50)
AMN of low grade	2 (20)
Adenocarcinoma well and poorly differentiated	2 (20)
Moderately differentiated NET	1 (10)
**HIPEC indication**	**3 (4.7)**
AMN of low grade	3 (100)
**Monitoring by endocrinology**	**5 (7.8)**
Well-differentiated NET	5 (100)
**Follow-up by oncology**	**2 (3.1)**
NET well-differentiated	1 (50)
AMN of low grade	1 (50)
**Patients who did not return to control**	**17 (26.5)**
AMN of low grade	8 (47.1)
NET well-differentiated	5 (29.4)
SSA dysplasia with high grade	4 (23.5)

SSA = sessile serrated adenoma, NET = neuroendocrine tumor, AMN = appendiceal mucinous neoplasm, RHC = right hemicolectomy, HIPEC = hyperthermic intraperitoneal chemotherapy

## Discussion

**Fig. 2:**
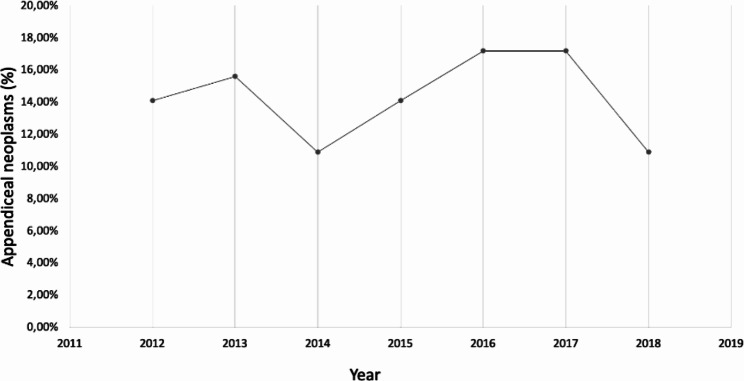
Distribution of appendiceal neoplasms per year

Our population shows that appendiceal tumors incidentally found in emergent appendectomies are significant, with an incidence of 2,14%, similar to those reported worldwide, between 0,2% and 2,5% [5, 11, 25]. From 2012 to 2018, an increase in the number of new cases of appendiceal neoplasms was evidenced (Fig. 2). It is a fact that appendiceal neoplasms are rare and non-frequent entities found incidentally in surgical specimens of appendectomies [5, 18], and the importance of studying these surgical specimens for detecting appendiceal tumors, and establishing histologic subtypes, prognosis, and further treatment becomes a priority [4, 10, 12, 15].

Currently, the treatment of acute appendicitis is controversial since most recent literature has shown that the conservative management of these patients with the use of antibiotics in selected cases, is a viable and promising non-invasive option [1, 5, 6]. Notwithstanding, the increasing incidence of appendiceal tumors has shown the importance and need to carry out an anatomopathological study of the resected sample from surgery [22].

Different studies evaluating the non-operative management of uncomplicated acute appendicitis, such as the APPAC [1] and the CODA trial [26] have proven safety and non-inferiority of such treatment and have confirmed the possibility of a non-surgical management option for acute appendicitis. Nevertheless, the percentage of appendiceal neoplasms in the group of patients who did not undergo appendectomy is unclear. Moreover, advanced stages of a possible appendiceal neoplasm could be more frequently presented due to not intervening surgically in these patients leading to increased morbidity and mortality in this specific group of patients [3, 8, 18, 19]. Nevertheless, it is still unclear if this difference is statistically significant.

M. Enblad et al. [3] showed that patients who underwent non-surgical management of acute appendicitis had an increase in the incidence of neoplasms of both the appendix and the right-sided colon in all age groups compared to the general population. In the case of right colon cancer, the increased incidence is lasting 5 years after appendicitis, except for the population under 20 years [3]. Furthermore, in cases in which signs and symptoms of appendicitis are still present even though a patient has been conservatively treated, the surgeon must raise an alert to rule out an underlying neoplasm that may be causing the symptoms, not detected previously by imaging because of diagnostic methods limitations [3, 27].

Appendicular inflammatory masses can be presented in 2 to 6% of cases, with a higher risk of appendiceal neoplasms that may vary between 5,2 and 12% [28, 29]. Moreover, these cases can be considered controversial since there is the possibility of non-surgical management [4]. Therefore, the surgical team must highly consider performing interval appendectomy [20]. Meanwhile, in this study, malignant tumors were the most common, followed by benign and premalignant lesions respectively. Most surgical procedures were performed by laparoscopy (98,4%). In the APPAC study [1], open appendectomies were more frequent, which could eventually have increased morbidity in the group of patients who underwent surgery.

Among the limitations of this study are the relatively small number of patients, its retrospective nature, and the lack of longer follow-up. Further prospective studies are needed to evaluate the impact of non-surgical management of appendiceal inflammatory processes in terms of morbidity and mortality of appendicular neoplasms.

## Conclusion

Tumors of the cecal appendix must be considered as differential diagnoses to be ruled out in patients with suspected acute appendicitis. Unfortunately, they do not present specific signs or symptoms and are not suspected or evidenced before, or during surgical intervention, so their diagnosis remains incidental after the anatomopathological analysis of the surgical samples obtained from appendectomies. Appendiceal tumors are infrequent. However, the increasing incidence reported in recent international series, including the current one, enhances the need for the surgeon to discard this pathology as a cause of acute appendicitis. The possibility of incidentally diagnosing appendiceal neoplasms should be considered when making decisions about the appropriate management for acute appendicitis, whether surgical or not, considering that the histopathological analysis of the surgical specimen should be used for diagnostic purposes and to determine the requirement for additional treatment.

### Electronic supplementary material

Below is the link to the electronic supplementary material.

Supplementary Material 1

## Data Availability

The datasets used and/or analyzed during the current study are available from the corresponding author upon reasonable request.

## Abbreviations

RHC: Right hemicolectomy
NET: Neuroendocrine tumor
AJCC: American Joint Committee of Cancer
AMN: Mucinous neoplasm of the appendix
SSA: **Sessile serrated adenoma**
APPAC: **The Appendicitis Acuta project**
HIPEC: Hyperthermic intraperitoneal chemotherapy
