# Supplementary material for: Incidence of appendiceal neoplasms in appendectomy patients
Source: BMC Surg. 2023 Sep 21;23:287. doi: 10.1186/s12893-023-02183-4 (PMC10512515; doi:10.1186/s12893-023-02183-4)
Supplement: Supplementary file 1 — Supplementary Material 1 [file 12893_2023_2183_MOESM1_ESM.pptx]

## Slide 1
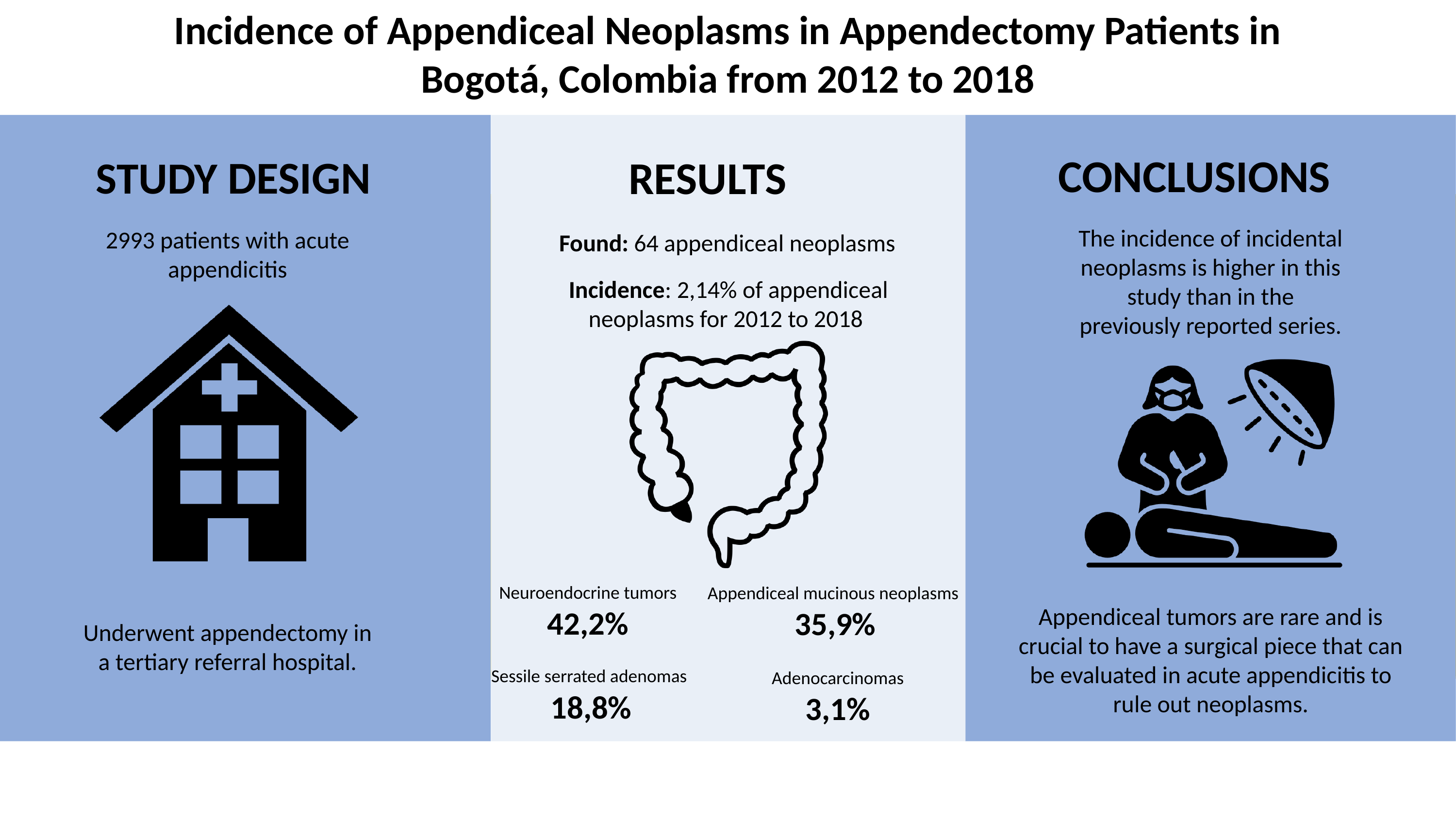

Incidence of Appendiceal Neoplasms in Appendectomy Patients in
Bogotá, Colombia from 2012 to 2018
CONCLUSIONS
STUDY DESIGN
RESULTS
The incidence of incidental neoplasms is higher in this study than in the previously reported series.
2993 patients with acute appendicitis
Found: 64 appendiceal neoplasms
Incidence: 2,14% of appendiceal neoplasms for 2012 to 2018
Neuroendocrine tumors 42,2%
Appendiceal mucinous neoplasms
35,9%
Appendiceal tumors are rare and is crucial to have a surgical piece that can be evaluated in acute appendicitis to rule out neoplasms.
Underwent appendectomy in a tertiary referral hospital.
Sessile serrated adenomas
18,8%
Adenocarcinomas
3,1%
